# Scale Factor Estimation for Quadrotor Monocular-Vision Positioning Algorithms

**DOI:** 10.3390/s22208048

**Published:** 2022-10-21

**Authors:** Alejandro Gómez-Casasola, Hugo Rodríguez-Cortés

**Affiliations:** Centro de Investigación y de Estudios Avanzados del Instituto Politécnico Nacional, Av. Instituto Politécnico Nacional 2508, Col. San Pedro Zacatenco, Ciudad de Mexico 07360, Mexico

**Keywords:** sensor fusion, quadrotor, observer design

## Abstract

Unmanned aerial vehicle (UAV) autonomous navigation requires access to translational and rotational positions and velocities. Since there is no single sensor to measure all UAV states, it is necessary to fuse information from multiple sensors. This paper proposes a deterministic estimator to reconstruct the scale factor of the position determined by a simultaneous localization and mapping (SLAM) algorithm onboard a quadrotor UAV. The position scale factor is unknown when the SLAM algorithm relies on the information from a monocular camera. Only onboard sensor measurements can feed the estimator; thus, a deterministic observer is designed to rebuild the quadrotor translational velocity. The estimator and the observer are designed following the immersion and invariance method and use inertial and visual measurements. Lyapunov’s arguments prove the asymptotic convergence of observer and estimator errors to zero. The proposed estimator’s and observer’s performance is validated through numerical simulations using a physics-based simulator.

## 1. Introduction

Nowadays, quadrotors are used in various applications, thanks to their low cost, mechanical robustness, and high maneuverability. Such applications include homeland security, forest-fire control, surveillance, sea and land exploration, human search and rescue, archaeological exploration, and volcanic activity monitoring, among many others [1,2]. Most of the abovementioned applications become impractical or even dangerous for human operators; thus, autonomous navigation, control, and guidance are required.

A quadrotor can perform autonomous navigation in unknown environments when its autopilot has access to all states. However, providing access to all quadrotor states without relying on a remote computer or sensors demands a vehicle with the capacity to process and extract information from only onboard sensors. The bottleneck to measuring all quadrotor states is that there are no out-of-the-box functional and reliable sensors to measure all states directly. For example, the quadrotor’s attitude is obtained by fusing the measurements from an inertial measurement unit (IMU). On the other hand, the global position system (GPS) is the primary sensor used for quadrotor positioning, but it presents limitations. It fails in environments where satellite communication is degraded, called GPS-denied environments, such as water bodies and indoors [3]. Furthermore, low-cost GPS does not provide enough resolution for trajectories on a centimeter scale, and the price of a GPS with higher resolution, such as differential GPS, increases drastically.

An algorithm to fuse GPS measurements with optical flow information using a Kalman filter (KF) was proposed in [4]. Shortly after, the work reported in [5] presented an improved sensor fusion algorithm based on an extended Kalman filter (EKF) that includes the measurements from an inertial navigation system (INS). Both sensor fusion algorithms improved the position estimation for low-cost GPS but not for GPS-denied environments.

Computer vision has emerged as a powerful solution for quadrotor position estimation. Visual sensors have many advantages over other sensors: they are cheap, provide color and geometric information for scene understanding, and consume less power. Many computer-vision algorithms are available for position estimation. For example, visual odometry (VO) estimates the ego-motion of a vehicle with an onboard camera. VO incrementally estimates the vehicle’s pose by examining the changes that motion induces on the input images [6]. Using a red, green, blue, depth (RGBD) camera, the method reported in [7] improved the VO algorithm by including a novel covariance estimation technique. The resulting VO-based algorithm allowed autonomous quadrotor navigation with satisfactory results. The depth camera measurement allows for determining the position scale factor.

The SLAM algorithm estimates the vehicle’s pose and, at the same time, constructs a map of the surroundings. The most successful versions of SLAM, running in real time, are ORB-SLAM [8] and LSD-SLAM [9]. These SLAM variants rely on techniques used to calculate the camera position and construct the map, such as feature extraction, or direct methods that operate on the image intensities [10]. A comparison between SLAM algorithms for mobile robot navigation in indoor environments is reported in [11], where it is concluded that ORB-SLAM can be used to determine the robot position with an additional module to recover the scale factor. The report [12] presents quadrotor autonomous navigation using a SLAM algorithm without determining the position scale factor.

Semidirect visual odometry (SVO) is a hybrid algorithm combining feature-based and direct methods. It estimates the relative motion between two frames by minimizing photometric errors. The projection error between the location of the feature points and their predicted positions is minimized to obtain the optimal camera pose [13]. The autonomous navigation of a UAV using SVO and a recovery mechanism to reinitialize the visual map when a failure occurs are proposed in [14]. The navigation strategy includes a pose estimation scheme for temporary vehicle control and a method to correct the scene scale factor using altitude measurements.

With some differences, computer-vision algorithms can be implemented using monocular or stereoscopic cameras. Stereo cameras can capture three-dimensional images, meaning the scene’s depth is known. This ability leads to better accuracy and resolution than in the monocular camera case for position estimation purposes. The algorithms ORB-SLAM, LSD-SLAM, and SVO, among others, were first developed for monocular camera implementation and, years after, improved for multi-camera configurations as reported in [15,16,17], respectively.

Nevertheless, monocular cameras are preferred for implementation in small vehicles for several reasons: a single camera is easy to mount due to its smaller size, is lighter and cheaper, and consumes less power. Additionally, a single camera configuration is free from the burden of multi-camera calibration and requires less processing power from the CPU onboard than multi-camera configurations [18]. Only one drawback is present for monocular cameras: they cannot recover the image’s three-dimensional structure and the camera position with complete metric information; in other words, the information on the scene’s depth is unavailable. This phenomenon is known as similarity ambiguity [19]. At least one piece of metric information is required to recover the absolute scale factor. This cue may come from prior scene knowledge, such as camera height, object size, vehicle speed, stereo camera baseline, or other sensors such as LiDAR or GPS.

Some methods have been proposed to deal with the similarity ambiguity problem. In [20,21], the extra piece of geometric information to determine the position measurement scale factor comes from an ultrasonic sensor and a one-dimensional laser range finder (LRF), respectively. These approaches require additional sensors onboard. Besides, the absolute scale is only calculated on the axis where the sensor is mounted, so it is assumed that the scale is the same on the other two axes, which is not always valid.

An EKF algorithm considering multirotor dynamics is proposed in [22] to estimate the scale factor online. A scale factor observability analysis supports the estimator design. However, using EKF on this approach makes the estimator nondeterministic, so stability is not formally proven.

In the field of deterministic estimators, ref. [23] presents a scale estimator based on control stability. It shows that the absolute scale and control gain have a unique linear relationship. The absolute scale can be estimated by detecting self-induced oscillations and analyzing the system stability. The problem with this approach is that an adaptive control technique must be used for an online estimation, leaving out other types of controllers.

This article presents a scale factor estimator in the cartesian plane fused with a velocity observer, deterministic and based on the quadrotor dynamic model, using only onboard sensors. The set of sensors provides the quadrotor’s attitude and angular velocity from an attitude and heading reference system (AHRS), the quadrotor’s acceleration from the set of sensors of the inertial measurement unit, and the scaled position from a SLAM algorithm based on a monocular camera. The scale factor estimator and the velocity observer are designed following the immersion and invariance methodology introduced in [24]. The singular contributions of this work are: the estimator and observer are designed considering the full quadrotor nonlinear model, the Coriolis forces are not neglected, the position scale factor is reconstructed in all three dimensions, and it is formally proven using Lyapunov arguments that the estimator and observer errors locally asymptotically converge to zero. Numerical simulations using Gazebo are presented to support the theoretical developments. The outcomes of this paper are based on the preliminary works reported in [25,26].

The remaining parts of the paper are arranged as follows. The sensor models used are presented in Section 2, along with the quadrotor dynamics. Section 3 outlines the fundamental contribution of this paper and describes the mathematical advancements used to create the scale factor estimator. Through numerical simulations, Section 4 illustrates the performance of the estimator. Finally, Section 5 wraps up this paper with a few closing thoughts and suggestions for future work.

## 2. Materials and Methods

Table 1 summarizes the notation used to introduce the quadrotor dynamic model.

In Table 1, the International System of Units is considered, and

$$SO\left(3\right)=\left\{R\in {\mathbb{R}}^{3\times 3}\;|\;R^{⊤}R=I,\;\det\left(R\right)=1\right\}$$

with *I* the identity matrix.

### 2.1. Quadrotor Dynamics

**Figure 1:**
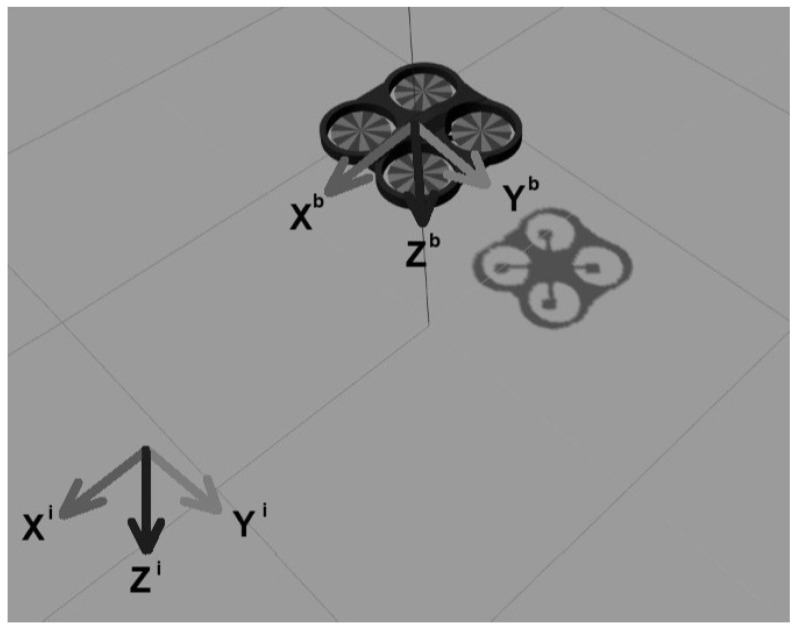
Inertial $X^{i}Y^{i}Z^{i}$ and Body $X^{b}Y^{b}Z^{b}$ coordinates.

An inertial coordinate frame and a non-inertial coordinate frame (body frame) attached to the quadrotor center of gravity are needed to describe the quadrotor dynamic, see Figure 1. The following equations, expressed in mixed inertial and body coordinates, describe the translational and rotational quadrotor dynamics [26]:

(1) $$\begin{array}{ccc} \dot{X} & = & RV^{b}\\ m{\dot{V}}^{b} & = & mgR^{⊤}e_{3}-T_{T}e_{3}-\mu HV^{b}-mS\left(\Omega \right)V^{b}\\ \dot{R} & = & RS(\Omega )\\ J\dot{\Omega } & = & -S\left(\Omega \right)J\Omega +M^{b} \end{array}$$

with

$$e_{3}=\left[\begin{array}{c} 0\\ 0\\ 1 \end{array}\right],\;H=\left[\begin{array}{ccc} 1 & 0 & 0\\ 0 & 1 & 0\\ 0 & 0 & 0 \end{array}\right],\;S\left(\Omega \right)=\left[\begin{array}{ccc} 0 & -r & q\\ r & 0 & -p\\ -q & p & 0 \end{array}\right]$$

### 2.2. Available Sensors

It is assumed that the quadrotor carries onboard a set of sensors that provide the following measurements.

#### 2.2.1. Scaled Position

The vehicle carries a monocular camera facing the horizontal plane and the necessary computer power to implement a monocular-vision algorithm to determine its scaled inertial position. Therefore, the following measurement is available

(2) $$\begin{array}{c} y_{1}=X_{s}=\left[\begin{array}{c} k_{x}x\\ k_{y}y\\ k_{z}z \end{array}\right]=\left[\begin{array}{ccc} k_{x} & 0 & 0\\ 0 & k_{y} & 0\\ 0 & 0 & k_{z} \end{array}\right]\left[\begin{array}{c} x\\ y\\ z \end{array}\right]=diag\left(K\right)X \end{array}$$

where $X_{s}$ is the scaled position delivered by the monocular SLAM vision algorithm and $K={\left[\begin{array}{ccc} k_{x} & k_{y} & k_{z} \end{array}\right]}^{⊤}$ is the dimensionless unknown scale factor on the axes $X^{i}Y^{i}Z^{i}$, respectively.

**Remark** **1.** *The operator diag(A) represents a diagonal matrix whose elements are the elements of vector $A\in {\mathbb{R}}^{3}$. This operator satisfies the following indentities*

(3) diag(A+B)=diag(A)+diag(B)

(4) diag(A)B=diag(B)A

*with $A,B\in {\mathbb{R}}^{3}$ vectors.*

#### 2.2.2. Specific Acceleration

Commonly, quadrotors are equipped with an inertial measuring unit (IMU) that measures the Earth’s magnetic field intensity, angular velocity, and specific acceleration in body coordinates. According to the Accelerometer Tutorial reported in [27], the specific acceleration measured by an accelerometer mounted on a quadrotor is given by

(5) $$a^{b}=\frac{1}{m}F_{T}^{b}-gR^{⊤}e_{3}$$

where $a^{b}$ is the specific acceleration measured in the body axis and $F_{T}^{b}$ is the total external force acting on the quadrotor expressed in the body axis. From the quadrotor dynamics model in Equation (1), it follows that

(6) $$F_{T}^{b}=mgR^{⊤}e_{3}-T_{T}e_{3}-\mu HV^{b}$$

as a result,

(7) $$a^{b}=-\frac{T_{T}}{m}e_{3}-\frac{\mu }{m}HV^{b}$$

Hence, the specific acceleration is an available output, element wise it reads as

(8) $$y_{2}=\left[\begin{array}{c} a_{x}^{b}\\ a_{y}^{b}\\ a_{z}^{b} \end{array}\right]=-\frac{1}{m}\left[\begin{array}{c} \mu u\\ \mu v\\ T_{T} \end{array}\right]$$

with $a_{x}^{b}$, $a_{y}^{b}$ and $a_{z}^{b}$ the specific acceleration along the body axis.

#### 2.2.3. Attitude and Heading Reference Systems

The device that computes the quadrotor’s attitude and rotational velocity from the IMU measurements is called the attitude and heading reference system (AHRS). Assuming that the quadrotor carries an AHRS, the following signals are available.

(9) $$\begin{array}{c} y_{3}=R=\left[\begin{array}{c} r_{1}^{⊤}\\ r_{2}^{⊤}\\ r_{3}^{⊤} \end{array}\right] \end{array}$$

(10) $$\begin{array}{c} y_{4}=\Omega =\left[\begin{array}{c} p\\ q\\ r \end{array}\right] \end{array}$$

where $r_{i}\in {𝕊}^{2}$, i=1,…,3 are the columns of the rotation matrix transposed,

$${𝕊}^{2}=\left\{A\in {\mathbb{R}}^{3}\;|\;A^{⊤}A=1\right\}$$

is the unit 2-sphere.

#### 2.2.4. Vertical Speed

Through the use of a laser sensor or an ultrasonic sensor, the vertical quadrotor position can be measured so that the vertical speed can be determined. As a result, it is presumed that the subsequent measurement is available

(11) $$y_{5}=w$$

Finally, note that the quadrotor translational dynamic, the first equation in (1), expressed in terms of the measured states reads as

(12) $${\dot{V}}^{b}=gy_{3}^{⊤}e_{3}+y_{2}-S\left(y_{4}\right)V^{b}$$

### 2.3. Immersion and Invariance Observers

The following developments are based on Chapter 5 of [24]. Consider the following non-linear, deterministic, time invariant system

(13) $$\begin{array}{ccc} \dot{\eta } & = & f_{1}\left(\eta ,y\right)\\ \dot{y} & = & f_{2}\left(\eta ,y\right) \end{array}$$

where $\eta \in 𝓡\subset {\mathbb{R}}^{n}$ and $y\in 𝓨\subset {\mathbb{R}}^{m}$ are the unmeasured and measured states, respectively. It is assumed that the vector fields $f_{1}\left(\eta ,y\right)$ and $f_{2}\left(\eta ,y\right)$ are forward complete.

**Definition** **1.** *The dynamic system*

(14) $$\begin{array}{ccc} \dot{\hat{\eta }} & = & \varphi (\hat{\eta },y) \end{array}$$

*with* **$\hat{\eta }\in {\mathbb{R}}^{n}$** *, is an observer for the unmeasured state η if there exists a mapping* **$\beta :{\mathbb{R}}^{n}\times {\mathbb{R}}^{m}\to {\mathbb{R}}^{n}$** *such that the manifold*

$$𝓜=\left\{\left(\eta ,\hat{\eta },y\right)\subset {\mathbb{R}}^{n}\times {\mathbb{R}}^{n}\times {\mathbb{R}}^{m}\;|\;\beta \left(\hat{\eta },y\right)=\eta \right\}$$

*has the following properties*

- *𝓜 is positively invariant,*
- *All trajectories of (13), (14) that start in a neighbourhood of 𝓜 asymptotically converge to 𝓜.*

The construction of the observer of the form given in Definition 1 requires additional properties on the mapping $\beta (\hat{\eta },y)$, as stated in the following result.

**Theorem** **1.** *Consider the system (13). Suppose that there exist differentiable maps $\beta :{\mathbb{R}}^{n}\times {\mathbb{R}}^{m}\to {\mathbb{R}}^{n}$ such that*

*A1*  *For all $\hat{\eta }$ and y the map $\beta (\hat{\eta },y)$ satisfies*
$$det\left(\frac{\partial \beta }{\partial \hat{\eta }}\right)\neq 0$$
*A2*  *The dynamic system*
(15) $$\dot{\tilde{\eta }}=f_{1}\left(\tilde{\eta }+\beta \left(\hat{\eta },y\right),y\right)-f_{1}\left(\beta \left(\hat{\eta },y\right),y\right)-\frac{\partial \beta }{\partial y}\left(f_{2}\left(\tilde{\eta }+\beta \left(\hat{\eta },y\right),y\right)-f_{2}\left(\beta \left(\hat{\eta },y\right),y\right)\right)$$
  *has a (globally) asymptotically stable equilibrium at $\tilde{\eta }=0$ uniformily in $\hat{\eta }$ and y.*

*Then, the system (14) with*

(16) $$\varphi \left(\hat{\eta },y\right)={\left(\frac{\partial \beta }{\partial \hat{\eta }}\right)}^{-1}\left(f_{1}\left(\beta \left(\hat{\eta },y\right),y\right)-\frac{\partial \beta }{\partial y}f_{2}\left(\beta \left(\hat{\eta },y\right),y\right)\right)$$

*is a (global) observer for (13).*

The proof of this Theorem is presented in Appendix A. The result expressed in Theorem 1 is followed to design the velocity observer and the scale factor estimator.

## 3. Observer and Estimator Design

This section discusses how the observer and the estimator that reconstruct $V^{b}$ and *K*, respectively, are designed from the available measurements of acceleration, scaled position, attitude, angular velocity, and vertical speed.

### 3.1. Observation and Estimation Problems

The following terms state the observation problem. Assume that the outputs $y_{i},$i=1,…,5 are measurable. Design two dynamic systems, likely, of the form

$$\begin{array}{ccc} {\dot{\hat{V}}}^{b} & = & \varphi_{1}\left({\hat{V}}^{b},y_{2},y_{3},y_{4},y_{5}\right)\\ \dot{\hat{K}} & = & \varphi_{2}\left(\hat{K},{\hat{V}}^{b},y_{1},y_{2},y_{3},y_{4},y_{5}\right) \end{array}$$

where ${\hat{V}}^{b}\in {\mathbb{R}}^{3}$ and $\hat{K}\in {\mathbb{R}}^{3}$, such that two functions exist, $\beta_{1}\in {\mathbb{R}}^{3}$ and $\beta_{2}\in {\mathbb{R}}^{3}$, that depend on the available information, and the following identities asymptotically hold

(17) $$\begin{array}{ccc} \lim_{t\to \infty }\beta_{1}\left({\hat{V}}^{b},y_{2},y_{3},y_{4},y_{5}\right) & = & V^{b}\\ \lim_{t\to \infty }\beta_{2}\left(\hat{K},{\hat{V}}^{b},y_{1},y_{2},y_{3},y_{4},y_{5}\right) & = & K \end{array}$$

### 3.2. Velocity Observer

According to the immersion and invariance technique, the observation error is defined as follows

(18) $${\tilde{V}}^{b}=V^{b}-\beta_{1}\left({\hat{V}}^{b},\sigma \right)$$

with $\beta_{1}$ element wise reading as

(19) $$\beta_{1}=\left[\begin{array}{c} \beta_{1x}\\ \beta_{1y}\\ \beta_{1z} \end{array}\right]$$

where

(20) $$\dot{\sigma }=\left[\begin{array}{c} a_{x}^{b}\\ a_{y}^{b}\\ -w \end{array}\right]=\left[\begin{array}{c} -\frac{\mu }{m}u\\ -\frac{\mu }{m}v\\ -w \end{array}\right]=-\bar{H}V^{b}$$

and

$$\bar{H}=\left[\begin{array}{ccc} \frac{\mu }{m} & 0 & 0\\ 0 & \frac{\mu }{m} & 0\\ 0 & 0 & 1 \end{array}\right]$$

Equation (18) models the distance to the manifold 𝓜 of Definition 1, where the velocity $V^{b}$ is equal to $\beta_{1}\left({\hat{V}}^{b},\sigma \right)$. This distance must asymptotically converge to zero to complete the observer design. Note that the output $y_{2}$ is not directly used since the time derivative of ${\tilde{V}}^{b}$ will require the computation of ${\dot{y}}_{2}$; this is the reason why the new state σ is introduced.

The time derivative of ${\tilde{V}}^{b}$ is given by

(21) $${\dot{\tilde{V}}}^{b}={\dot{V}}^{b}-\frac{\partial \beta_{1}}{\partial {\hat{V}}^{b}}{\dot{\hat{V}}}^{b}-\frac{\partial \beta_{1}}{\partial \sigma }\dot{\sigma }$$

Substituting ${\dot{V}}^{b}$, the body velocity $V^{b}$ and the time derivative of σ from Equations (12), (18), and (20), respectively, one has

(22) $$\begin{array}{c} {\dot{\tilde{V}}}^{b}=gy_{3}^{⊤}e_{3}+y_{2}-S\left(y_{4}\right)\left({\tilde{V}}^{b}+\beta_{1}\right)-\frac{\partial \beta_{1}}{\partial {\hat{V}}^{b}}{\dot{\hat{V}}}^{b}+\frac{\partial \beta_{1}}{\partial \sigma }\bar{H}\left({\tilde{V}}^{b}+\beta_{1}\right) \end{array}$$

Now, the observer state dynamic ${\dot{\hat{V}}}^{b}$ is defined in terms of the known signals, as

(23) $$\begin{array}{c} {\dot{\hat{V}}}^{b}={\frac{\partial \beta_{1}}{\partial {\hat{V}}^{b}}}^{-1}\left(gy_{3}^{⊤}e_{3}+y_{2}-S\left(y_{4}\right)\beta_{1}+\frac{\partial \beta_{1}}{\partial \sigma }\bar{H}\beta_{1}\right) \end{array}$$

Substituting (23) into (22), one obtains

(24) $${\dot{\tilde{V}}}^{b}=-S\left(y_{4}\right){\tilde{V}}^{b}+\frac{\partial \beta_{1}}{\partial \sigma }\bar{H}{\tilde{V}}^{b}$$

Consider the following Lyapunov function to define the function $\beta_{1}\left({\hat{V}}^{b},\sigma \right)$

(25) $$\nu_{V}=\frac{1}{2}{\tilde{V}}^{b}{}^{⊤}{\tilde{V}}^{b}$$

it follows that

(26) $${\dot{\nu }}_{V}={\tilde{V}}^{b}{}^{⊤}\frac{\partial \beta_{1}}{\partial \sigma }\bar{H}{\tilde{V}}^{b}$$

Hence, to guarantee that the time derivative of $\nu_{V}$ is negative-definite, the matrix $\frac{\partial \beta_{1}}{\partial \sigma }\bar{H}$ must also be negative-definite. On the other hand, Equation (23) requires the matrix $\frac{\partial \beta_{1}}{\partial {\hat{V}}^{b}}$ to be invertible. Note that selecting

(27) $$\beta_{1}\left({\hat{V}}^{b},\sigma \right)={\hat{V}}^{b}-\Gamma_{v}\sigma$$

with

(28) $$\Gamma_{v}=\left[\begin{array}{ccc} \gamma_{vx} & 0 & 0\\ 0 & \gamma_{vy} & 0\\ 0 & 0 & \gamma_{vz} \end{array}\right]$$

and $\gamma_{vx}$, $\gamma_{vy}$ and $\gamma_{vz}$ positive gains, it follows that

(29) $${\dot{\nu }}_{V}=-{\tilde{V}}^{b}{}^{⊤}\Gamma_{v}\bar{H}{\tilde{V}}^{b}=-{\tilde{V}}^{b}{}^{⊤}diag\left(h\right){\tilde{V}}^{b},\;\frac{\partial \beta_{1}}{\partial {\hat{V}}^{b}}=I_{3}$$

with $h={\left[\begin{array}{ccc} \frac{\gamma_{vx}\mu }{m} & \frac{\gamma_{vy}\mu }{m} & \gamma_{vz} \end{array}\right]}^{⊤}$ and $I_{3}$ the 3×3 identity matrix. As a result, $\frac{\partial \beta_{1}}{\partial \sigma }\bar{H}$ is negative-definite and $\frac{\partial \beta_{1}}{\partial {\hat{V}}^{b}}$ is invertible.

### 3.3. Scale Factor Estimator

In reference [25], the scale factor estimator needs the translational velocity as a measurable output, but in this work it is available through the observer designed (27) according to (17). It is important to note also in this equation that $\beta_{2}$ depends on states expressed in mixed inertial and body coordinates, unlike $\beta_{1}$ which depends only on states expressed in body coordinates. In order to have all the states expressed in inertial coordinates, $\beta_{1}$ needs to be translated with the rotation matrix. The inertial velocity is introduced as follows

(30) $$V^{i}=y_{3}V^{b}$$

Additionally, the inertial velocity observer error is defined

(31) $${\tilde{V}}^{i}=V^{i}-{\hat{V}}^{i}$$

with

(32) $${\hat{V}}^{i}=y_{3}\beta_{1}$$

Now, the scale factor estimation error is defined in the following form

(33) $$\tilde{K}=K-\beta_{2}\left(\hat{K},y_{1},{\hat{V}}^{i}\right)$$

with $\beta_{2}$ element wise reading as

(34) $$\beta_{2}=\left[\begin{array}{c} \beta_{2x}\\ \beta_{2y}\\ \beta_{2z} \end{array}\right]$$

The derivative with respect to the time of the estimation error is

(35) $$\dot{\tilde{K}}=-\frac{\partial \beta_{2}}{\partial \hat{K}}\dot{\hat{K}}-\frac{\partial \beta_{2}}{\partial y_{1}}{\dot{y}}_{1}-\frac{\partial \beta_{2}}{\partial {\hat{V}}^{i}}{\dot{\hat{V}}}^{i}$$

From Equations (1) and (2), it follows that

(36) $$\begin{array}{ccc} {\dot{y}}_{1} & = & diag\left(K\right)y_{3}V^{b}=diag\left(K\right)V^{i}\\ {\dot{y}}_{3} & = & y_{3}S\left(y_{4}\right) \end{array}$$

By combining Equations (18) and (32), one obtains

(37) $$\begin{array}{ccc} {\hat{V}}^{i} & = & y_{3}\left(V^{b}-{\tilde{V}}^{b}\right) \end{array}$$

thus,

(38) $$\begin{array}{ccc} {\dot{\hat{V}}}^{i} & = & ge_{3}+y_{3}y_{2}-\Gamma_{v}\bar{H}{\tilde{V}}^{i} \end{array}$$

Substituting (36) and (37) into (35), one obtains

(39) $$\begin{array}{c} \dot{\tilde{K}}=-\frac{\partial \beta_{2}}{\partial \hat{K}}\dot{\hat{K}}-\frac{\partial \beta_{2}}{\partial y_{1}}diag\left(K\right)V^{i}-\frac{\partial \beta_{2}}{\partial {\hat{V}}^{i}}\left(ge_{3}+y_{3}y_{2}-\Gamma_{v}\bar{H}{\tilde{V}}^{i}\right) \end{array}$$

Now, substituting *K* from (33) and $V^{i}$ from (31), the scale factor estimation error becomes

(40) $$\dot{\tilde{K}}=-\frac{\partial \beta_{2}}{\partial \hat{K}}\dot{\hat{K}}-\frac{\partial \beta_{2}}{\partial y_{1}}diag\left(\tilde{K}+\beta_{2}\right)\left({\tilde{V}}^{i}+{\hat{V}}^{i}\right)-\frac{\partial \beta_{2}}{\partial {\hat{V}}^{i}}\left(ge_{3}+y_{3}y_{2}-\Gamma_{v}\bar{H}{\tilde{V}}^{i}\right)$$

The dynamic of the scale factor estimator state is defined in terms of the known signals as follows

(41) $$\begin{array}{c} \dot{\hat{K}}={\frac{\partial \beta_{2}}{\partial \hat{K}}}^{-1}\left(-\frac{\partial \beta_{2}}{\partial y_{1}}diag\left(\beta_{2}\right){\hat{V}}^{i}-\frac{\partial \beta_{2}}{\partial {\hat{V}}^{i}}\left(ge_{3}+y_{3}y_{2}\right)\right) \end{array}$$

After substituting $\dot{\hat{K}}$ into the scale factor estimator error (40), it follows that

(42) $$\begin{array}{c} \dot{\tilde{K}}=-\frac{\partial \beta_{2}}{\partial y_{1}}\left(diag\left(\tilde{K}\right){\hat{V}}^{i}+diag\left(\tilde{K}+\beta_{2}\right){\tilde{V}}^{i}\right)+\frac{\partial \beta_{2}}{\partial {\hat{V}}^{i}}\Gamma_{v}\bar{H}{\tilde{V}}^{i} \end{array}$$

Once again, the function $\beta_{2}$ needs to be defined to ensure that the estimation error $\tilde{K}$ converges to zero with $\frac{\partial \beta_{2}}{\partial \hat{K}}$ an invertible matrix. Thus, the following vector function is proposed

(43) $$\beta_{2}\left(\hat{K},y_{1},{\hat{V}}^{i}\right)=\hat{K}+\Gamma_{k}diag\left(y_{1}\right){\hat{V}}^{i}=\hat{K}+\Gamma_{k}diag\left({\hat{V}}^{i}\right)y_{1}$$

with

(44) $$\Gamma_{k}=\left[\begin{array}{ccc} \gamma_{kx} & 0 & 0\\ 0 & \gamma_{ky} & 0\\ 0 & 0 & \gamma_{kz} \end{array}\right]$$

and $\gamma_{ki},i=x,y,z$ the scale factor estimator gains.

Replacing (43) into (42), one obtains

(45) $$\begin{array}{c} \dot{\tilde{K}}=-\Gamma_{k}diag\left({\hat{V}}^{i}\right)\left(diag\left({\hat{V}}^{i}\right)\tilde{K}+diag\left({\tilde{V}}^{i}\right)K\right)+\Gamma_{k}diag\left(y_{1}\right)\Gamma_{v}\bar{H}{\tilde{V}}^{i} \end{array}$$

where (3), (4) and (33) had been considered. From (31), it follows that

(46) $$\begin{array}{ccc} \dot{\tilde{K}} & = & -\Gamma_{k}\left(diag\left(V^{i}\right)diag\left(V^{i}\right)+diag\left({\tilde{V}}^{i}\right)diag\left({\tilde{V}}^{i}\right)\right)\tilde{K}\\ & & +2\Gamma_{k}diag\left(V^{i}\right)diag\left({\tilde{V}}^{i}\right)\tilde{K}-\Gamma_{k}\left(diag\left(V^{i}\right)-diag\left({\tilde{V}}^{i}\right)\right)diag\left({\tilde{V}}^{i}\right)K\\ & & +\Gamma_{k}diag\left(y_{1}\right)\Gamma_{v}\bar{H}{\tilde{V}}^{i} \end{array}$$

The following assumptions are considered to state the main result of this paper.

**Assumption** **1.** *The following identity holds.*

$$\lim_{t\to \infty }\int_{0}^{t}diag\left(V^{i}\left(\tau \right)\right)diag\left(V^{i}\left(\tau \right)\right)d\tau =\infty I$$

*with $I\in {\mathbb{R}}^{3\times 3}$ the identity matrix.*

**Assumption** **2.** *There exist control inputs $T_{T}$ and $M^{b}$ such that the following quadrotor states can be upper bounded, this is*

(47) $$\begin{array}{c} ∥V^{i}∥\leq \kappa_{0},\;∥y_{1}∥\leq \kappa_{1} \end{array}$$

*for some not-necessarily constants $\kappa_{0}$ and $\kappa_{1}$. The notation ∥(·)∥ stands for the Euclidean norm for a matrix or vector (·).*

**Remark** **2.** *Assumption 1 is the persistence of the excitation condition; in this case, fulfilling this condition implies that the quadrotor must move to estimate the scale factor successfully. Assumption 2 means that a control loop allows the quadrotor to fly stably; consequently, the quadrotor dynamics is forward complete.*

The following Proposition summarizes the main result of this work.

**Proposition** **1.** *Under Assumptions 1 and 2, there are matrix gains $\Gamma_{v}$ and $\Gamma_{k}$ such that the dynamic systems (23) and (41) are local observers for the translational velocity and estimators of the scale factor. The translational velocity and scaling factor are rebuilt in (27) and (43).*

The proof of this Proposition is reported in Appendix B.

## 4. Numerical Simulations

A numerical simulation study was performed on different platforms to evaluate the observer and estimator’s performance.

### 4.1. Matlab-Simulink

The first one was performed using Matlab-Simulink, to avoid problems such as sensors’ noise and external disturbances so that we can evaluate the estimators by themselves. A program was designed to simulate a quadrotor in a closed loop with the controller developed in [28], tracking a circular trajectory on the Cartesian plane and a sinusoidal form on the vertical plane. To fulfill Assumption 1, the desired trajectories were $x_{d}=Acos\left(\omega t\right)$, $y_{d}=Asin\left(\omega t\right)$ and $z_{d}=Acos\left(\omega t\right)$. For the velocity observer, the initial conditions used were $V^{b}\left(0\right)={\left[\begin{array}{ccc} -0.2 & 0.3 & 0.2 \end{array}\right]}^{⊤}$ with a proposed μ=0.6 and gains $\gamma_{vx}=1.2$, $\gamma_{vy}=1.2$ and $\gamma_{vz}=1.2$. Regarding the monocular-vision positioning algorithm for this numerical simulation, any real values for the scale factor *K* can be used; nevertheless, in more realistic simulations such as the ones in the next section, it is observed that the scaled position is always smaller than the real position, $X_{s}<X$, so K<1; hence, the real values of the scale factor for this simulation where fixed at $K={\left[0.65\;\;0.7\;\;0.55\right]}^{⊤}$. The gains used for the estimator were $\gamma_{kx}=2.0$, $\gamma_{ky}=2.0$ and $\gamma_{kz}=2.0$.

**Figure 2:**
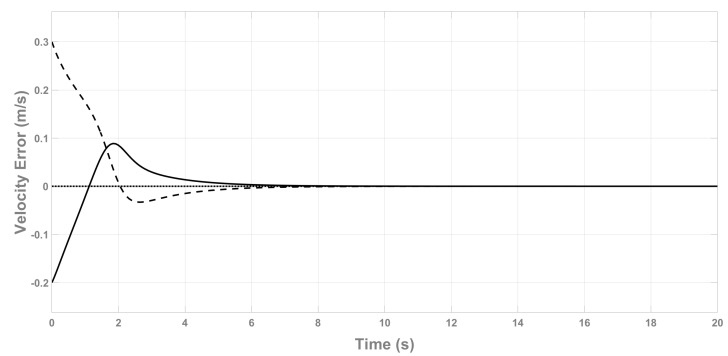
Velocity observer error ${\tilde{V}}^{b}$. $\tilde{u}$ (continuous line), $\tilde{v}$ (dashed line), $\tilde{w}$ (dotted line).

Figure 2 shows the velocity observer error ${\tilde{V}}^{b}$ for this simulation, where it can be seen that the velocities converge to zero correctly. Note that the velocity error on the *Z* axis, $\tilde{w}$, is always zero because the speed on this axis is measurable, (11).

**Figure 3:**
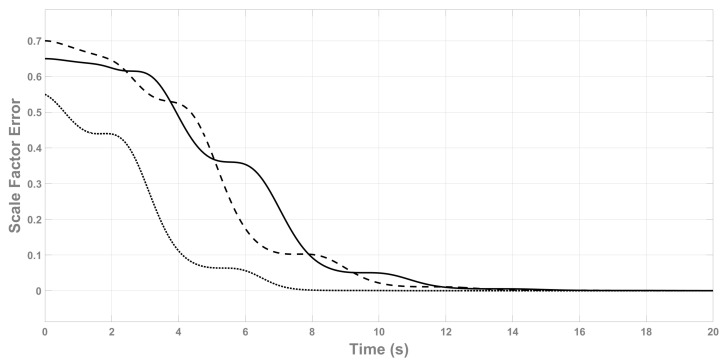
Scale factor estimator error $\tilde{K}$. ${\tilde{k}}_{x}$ (continuous line), ${\tilde{k}}_{y}$ (dashed line), ${\tilde{k}}_{z}$ (dotted line).

Figure 3 shows the scale factor estimator error $\tilde{K}$ that also proves the correct convergence of the estimator. In this graph the cascade behavior of (46) with (24) can also be seen, which means that $\dot{\tilde{K}}$ will always converge after ${\dot{\tilde{V}}}^{b}$ due to the interconnection term Ψ.

### 4.2. Gazebo

It is time to put the observers to the test in a simulator, such as Gazebo, which is more like reality after confirming their proper operation in a controlled setting. The Gazebo is an open-source 3D robotics simulator. It incorporates the open dynamics engine (ODE) as a physics engine, OpenGL for graphics, support code for sensor simulation, and actuator control. The robot operating system (ROS) and the Gazebo simulator are wholly linked.

In the previous simulation, it was easy to set simulation values for the constant μ and the scaled position $X_{s}$ delivered by a monocular-vision algorithm. In the Gazebo simulation, such values will have to be treated more rigorously as would be performed in an actual experiment.

#### 4.2.1. Calculation of μ

μ is a term related to the drag coefficient, a positive constant representing a combination of the profile and induced drag forces on the rotors, known in the helicopter literature as “rotor drag” [27,29]. Like any other parameter of the quadrotor, such as its weight (*m*), it must be measured before experiments. Due to the units of this parameter, listed in Table 1, it is known as the “mass flow rate”.

From Equation (8), it can be seen that

(48) $$\begin{array}{ccc} a_{x}^{b} & = & -\frac{\mu }{m}u\\ a_{y}^{b} & = & -\frac{\mu }{m}v \end{array}$$

From any of these two equations, the constant μ can be measured by having access to the accelerometer and translational velocities measurements, for example

(49) $$\begin{array}{ccc} \mu & = & -\frac{a_{x}^{b}}{u}m \end{array}$$

We have access to both of these measurements in Gazebo, so a circular trajectory tracking simulation was performed to measure these states. The calculated value for the quadrotor used in Gazebo is μ=0.18.

**Figure 4:**
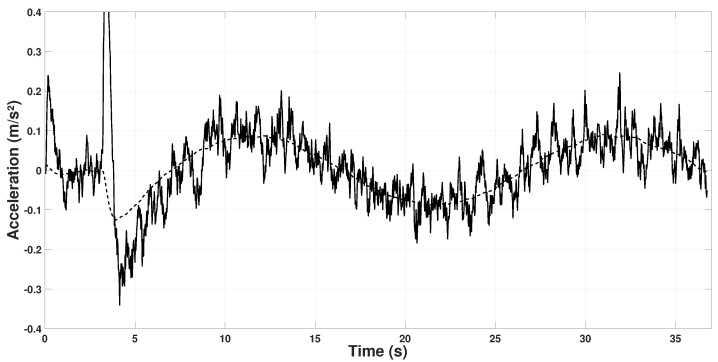
Relation between accelerometer and μ on the $X^{b}$ axis. $a_{x}^{b}$ (continuous line), $-\frac{\mu }{m}u$ (dashed line).

With μ calculated, Figure 4 shows the Equation (48) on the *X* axis with the quadrotor following a circular trajectory, where it can be seen that the relation holds, so the calculated μ is correct. Note that the accelerometer readings ($a_{x}^{b}$) have noise added due to the rotors.

#### 4.2.2. Monocular-Vision Algorithm

As mentioned before, the designed scale factor estimator works with any computer-vision algorithm that implements a monocular camera and delivers position measurements. For this simulation, we use ORB-SLAM2 [15] due to its precision and accuracy, it is easy to install and well-documented, and it has ROS integration. Hence, it is easy to add to the Gazebo environment.

#### 4.2.3. Trajectory Tracking Control Using the Scale Factor Estimator

The simulation involves flying the quadrotor running ORB-SLAM2 from the visual information obtained by its onboard monocular camera without position control following a diagonal trajectory in the horizontal plane to obtain the information required to make the identities in (17) hold. After this step, the scale factor *K* will be known through $\beta_{2}$, so the quadrotor will be able to measure its actual position with $y_{1}$, (2). Then, the quadrotor will follow a lemniscate trajectory autonomously (closed-loop).

The low-level controller of the quadrotor is driven by [30], which is a driver to interface with Parrot AR-Drone quadrotors through ROS. It takes translational velocities as control inputs.

**Figure 5:**
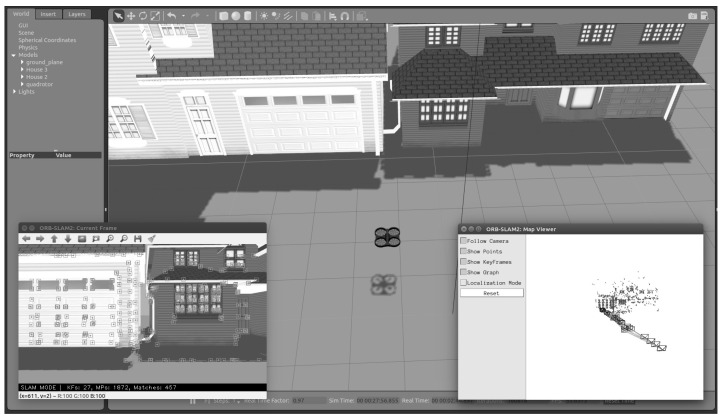
Gazebo environment.

Figure 5 shows the Gazebo environment with the SLAM algorithm running. The window on the left shows what the onboard camera is seeing with the features (points) detected, and the window on the right depicts the mapping construction.

The first step is to fly the quadrotor in open-loop over a diagonal trajectory from position (0,0) to (3,3), after that to the position (−3,−3) and finally back to the origin (0,0), always maintaining a constant velocity of $u_{d}=0.2$ and $v_{d}=0.2$.

**Figure 6:**
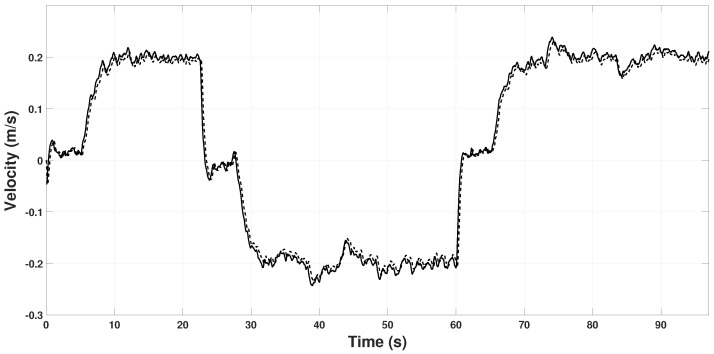
Observed speed on the $X^{b}$ axis. *u* (continuous line), $\beta_{1x}$ (dashed line).

**Figure 7:**
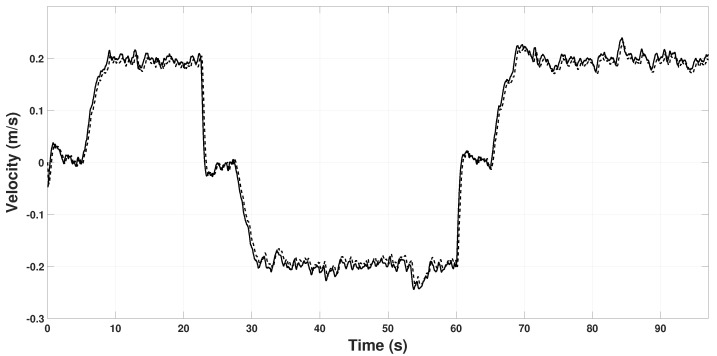
Observed speed on the $Y^{b}$ axis. *v* (continuous line), $\beta_{1y}$ (dashed line).

Figure 6 and Figure 7 show the real velocity and the estimated by the observer. With the initial conditions of the real velocity and the observer being the same, $V^{b}\left(0\right)={\left[0\;\;0\;\;0\right]}^{⊤}$, and the high gains $\gamma_{vx}=30$, $\gamma_{vy}=30$ and $\gamma_{vz}=30$, make the observer converge immediately.

**Figure 8:**
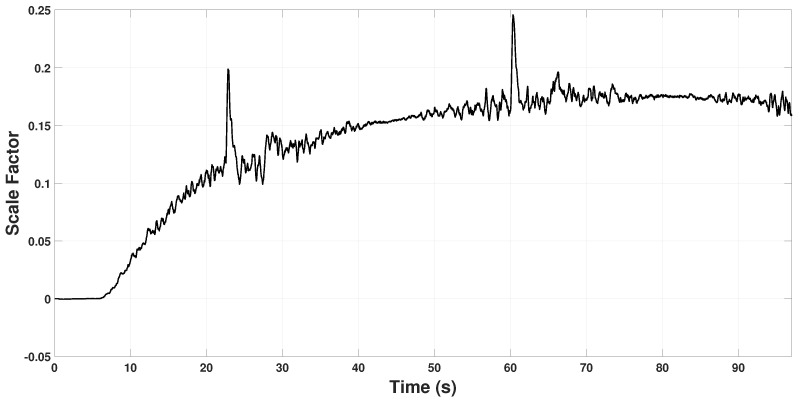
Estimated scale factor $\beta_{2x}$ on the $X^{i}$ axis.

**Figure 9:**
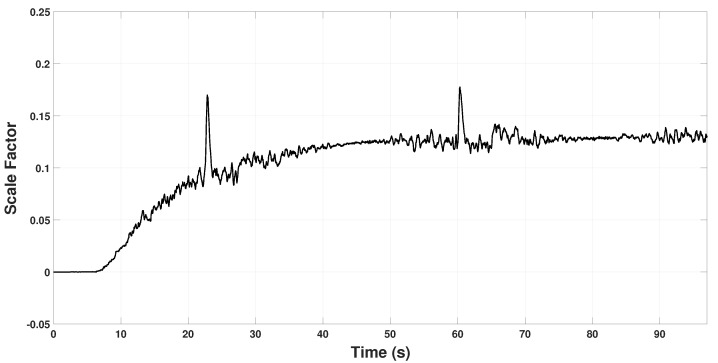
Estimated scale factor $\beta_{2y}$ on the $Y^{i}$ axis.

Figure 8 and Figure 9 show the scale factor estimation on the *X* and *Y* axes, respectively. Note that from time 0 s to 6 s, the estimator remains in zero because, in that period, the quadrotor takes off and holds in hover for some seconds, so Assumption 1 is not fulfilled. In the period 22 s to 60 s, some peaks appear because the quadrotor reaches the coordinates (3,3) and (−3,−3), respectively. Hence, it changes velocity abruptly to change its direction of movement to reach the next point. These changes in velocity can also be seen in Figure 6 and Figure 7 in the time periods.

After the open-loop routine, $\beta_{2}$ is calculated to converge at $k_{x}=0.175$ and $k_{y}=0.13$. It can also be seen in Figure 8 and Figure 9 that $\tilde{K}$ converges exponentially, as it was anticipated in (A7). From this point, knowing the scale factor *K*, the quadrotor will be able to measure its actual position through $y_{1}$ in (2) as follows

(50) $$X=diag{\left(K\right)}^{-1}y_{1}$$

**Figure 10:**
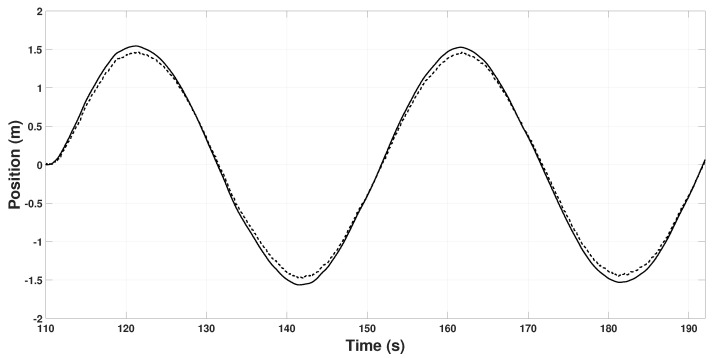
Quadrotor position on the $X^{i}$ axis. *x* (continuous line), $\frac{x_{s}}{k_{x}}$ (dashed line).

**Figure 11:**
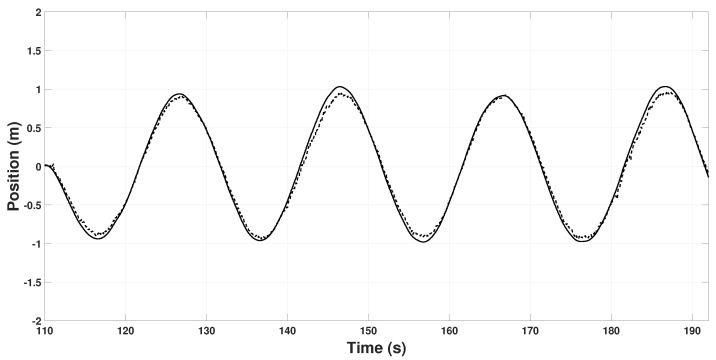
Quadrotor position on the $Y^{i}$ axis. *y* (continuous line), $\frac{y_{s}}{k_{y}}$ (dashed line).

Finally, using the estimated information, the quadrotor will follow a lemniscate trajectory autonomously in closed-loop; that is, $x_{d}=1.5\cos\left(\omega t\right)$ and $y_{d}=\sin\left(2\omega t\right)$. Figure 10 and Figure 11 show the real position and the position measured by the quadrotor on the *X* and *Y* axis, respectively, along the lemniscate trajectory.

**Figure 12:**
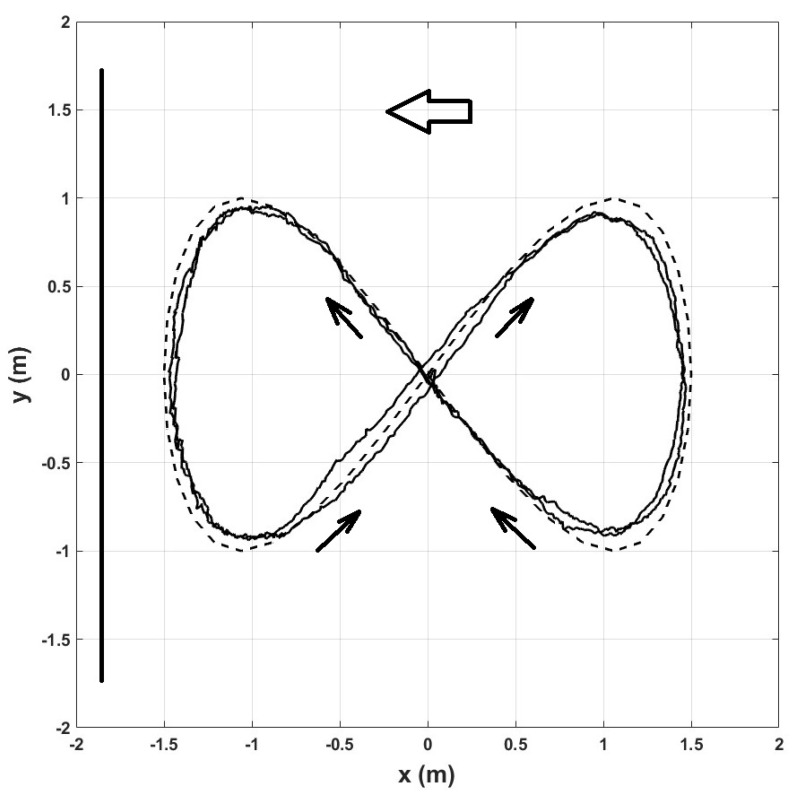
Lemniscate trajectory tracking using the scale factor estimator. Real position (continuous line), desired position (dashed line).

Figure 12 shows the XY graph of the trajectory validating the closed-loop control of the quadrotor using the proposed scale factor estimator. The quadrotor completed the whole lemniscate trajectory twice with the given simulation time. The big arrow indicates the direction the onboard camera faces the whole trajectory, the line on the left represents the buildings’ location, and the small arrows indicate the quadrotor motion direction.

As shown in Figure 12, the SLAM algorithm has better precision in the left part of the lemniscate because the camera is closer to the buildings, so more image features fed the algorithm. On the diagonals, the SLAM algorithm performs better when the quadrotor moves towards the buildings than when it moves away. Although the scale factor estimator recovers the actual position dimension, it does not help in any sense to improve the SLAM algorithm performance.

**Figure 13:**
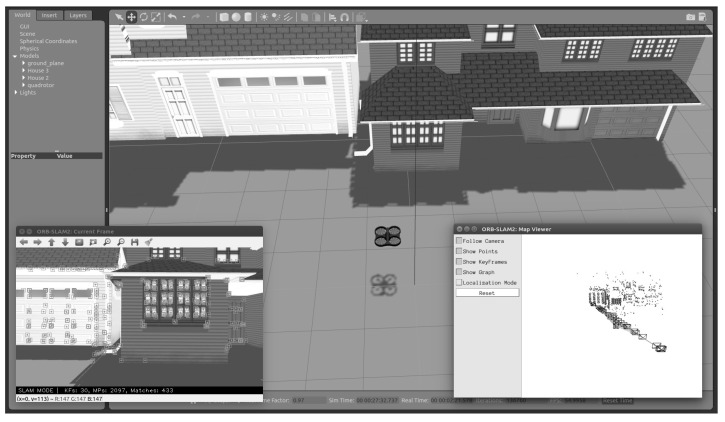
Gazebo environment with different initial conditions.

Changing the position of the house on the right in the Gazebo environment, we ran a second simulation to check if small changes in the initial conditions of the monocular-vision algorithm influence the final value of the scale factor. In this case, the house on the right is closer to the quadrotor, as shown in Figure 13. At the end of the simulation, the calculated scale factor values were $k_{x}=0.232$ and $k_{y}=0.166$, proving that the scale factor is different even for the same environment but with minor changes in the initial conditions.

## 5. Conclusions

This article proposed a velocity observer in cascade with a scale factor estimator. The significant contributions of this work are listed next:

- The velocity observer does not neglect the Coriolis term, offering greater accuracy in fast flights.
- The scale factor estimator allows taking advantage of all the benefits of monocular cameras, obtaining the accuracy of a stereoscopic camera without increasing the processing power.
- Lyapunov’s arguments prove asymptotic convergence to zero of the observer and estimator errors, and the simulations validate the correct performance and use of the proposed theory.
- It is illustrated that the scale factor is not the same in all axes, as some authors assume. It is even different from experimenting in the same environment if the initial conditions change.
- The proposed approach allows for position trajectory tracking to be performed directly using the measurements of a monocular-vision positioning algorithm, removing the limitations of a GPS or a motion capture system.

In future work, experiments will be carried out by a real quadrotor in more complex environments, combined with other kinds of computer vision algorithms such as person recognition or obstacle avoidance.

## Figures and Tables

**Table 1:** Quadrotor dynamic model notation.

Symbol	Variable	Units
$X={\left[\begin{array}{ccc} x & y & z \end{array}\right]}^{⊤}$	Translational position in inertial coordinates	m
R∈SO(3)	Rotation matrix from body to inertial coordinates	dimensionless
$V^{b}={\left[\begin{array}{ccc} u & v & w \end{array}\right]}^{⊤}$	Translational velocity in body frame coordinates	m/s
$\Omega ={\left[\begin{array}{ccc} p & q & r \end{array}\right]}^{⊤}$	Angular velocity in body coordinates	rad/s
$M^{b}={\left[\begin{array}{ccc} M_{x}^{b} & M_{y}^{b} & M_{z}^{b} \end{array}\right]}^{⊤}$	Moments generated by the differential thrust, and reaction moment between the four rotors	Nm
*m*	Quadrotor mass	kg
*g*	Gravity acceleration constant	m/s${}^{2}$
$T_{T}$	Total thrust generated by the four rotors	N
μ	Parameter related to aerodynamic drag force [27]	kg/s
$J\in {\mathbb{R}}^{3\times 3}$	Quadrotor inertia matrix	kg m${}^{2}$

## Data Availability

The simulation files are available upon request to the first author.

## Appendix A. Proof of Theorem 1

**Proof.** The distance of the trajectories of systems (13) and (14) to the set 𝓜 is characterized by

$$\tilde{\eta }=\eta -\beta \left(\hat{\eta },y\right)$$

The $\tilde{\eta }$ dynamic is given by

(A1) $$\dot{\tilde{\eta }}=f_{1}\left(\eta ,y\right)-\frac{\partial \beta }{\partial \hat{\eta }}\varphi \left(\hat{\eta },y\right)-\frac{\partial \beta }{\partial y}f_{2}\left(\eta ,y\right)$$

thus, replacing $\varphi (\hat{\eta },y)$ from Equation (16) into (A1), it follows that the $\tilde{\eta }$ dynamic becomes equal to (15). Finally, assumption A2 implies that (globally)

(A2) $$\lim_{t\to \infty }\beta \left(\hat{\eta },y\right)=\eta$$

□

## Appendix B. Proof of Proposition 1

**Proof.** The proof is divided into the following steps. In the first step, it is shown that the velocity observer error converges exponentially to zero. Then, it is proven that the trajectories of the estimation error do not explode in finite time. Finally, it is verified that the interconnection term between the observer and estimator dynamics satisfies a linear growth condition to the estimator error. Note that considering Equation (27), the time derivative of the Lyapunov function (26) can be written as follows

(A3) $${\dot{\nu }}_{V}=-{\tilde{V}}^{b}{}^{⊤}\Gamma_{v}\bar{H}{\tilde{V}}^{b}$$

as a result, one has ${\tilde{V}}^{b}$ converges exponentially to zero. Now, to prove that the estimation error trajectories do not explode in finite time, consider the following Lyapunov function

(A4) $$\nu_{K}=\frac{1}{2}{\tilde{K}}^{⊤}\tilde{K}$$

whose derivative with respect to time along the estimator dynamic (46) is

(A5) $$\begin{array}{ccc} {\dot{\nu }}_{K} & = & -{\tilde{K}}^{⊤}\Gamma_{k}\left(diag\left(V^{i}\right)diag\left(V^{i}\right)+diag\left({\tilde{V}}^{i}\right)diag\left({\tilde{V}}^{i}\right)\right)\tilde{K}\\ & & +2{\tilde{K}}^{⊤}\Gamma_{k}diag\left(V^{i}\right)diag\left({\tilde{V}}^{i}\right)\tilde{K}\\ & & -{\tilde{K}}^{⊤}\Gamma_{k}\left(diag\left(V^{i}\right)-diag\left({\tilde{V}}^{i}\right)\right)diag\left({\tilde{V}}^{i}\right)K\\ & & +{\tilde{K}}^{⊤}\Gamma_{k}diag\left(y_{1}\right)\Gamma_{v}\bar{H}{\tilde{V}}^{i} \end{array}$$

thus, ($\lambda_{M}\left(\cdot \right)$ stands for the maximal eigenvalue of matrix ·.)

(A6) $$\begin{array}{ccc} {\dot{\nu }}_{K} & \leq & \lambda_{M}\left(\Gamma_{k}\right)\left(∥diag\left(V^{i}\right){∥}^{2}+{∥diag\left({\tilde{V}}^{i}\right)∥}^{2}\right){∥\tilde{K}∥}^{2}\\ & & +2\lambda_{M}\left(\Gamma_{k}\right)∥diag\left(V^{i}\right)∥∥diag\left({\tilde{V}}^{i}\right)∥∥\tilde{K}{∥}^{2}\\ & & +\lambda_{M}\left(\Gamma_{k}\right)\left(∥diag\left(V^{i}\right)∥+∥diag\left({\tilde{V}}^{i}\right)∥\right)∥diag\left({\tilde{V}}^{i}\right)∥∥K∥∥\tilde{K}∥\\ & & +\lambda_{M}\left(\Gamma_{k}\right)∥diag\left(y_{1}\right)∥\lambda_{M}\left(\Gamma_{v}\right)\lambda_{M}\left(\bar{H}\right)∥{\tilde{V}}^{i}∥∥\tilde{K}∥ \end{array}$$

Hence, since the quadrotor dynamics is forward complete, the relationship in (A6) implies that the dynamic of $\tilde{K}$ does not blow up in finite time. Note that if the error signal ${\tilde{V}}^{b}$ is equal to zero the estimation error dynamic (46) reduces to

(A7) $$\begin{array}{ccc} \dot{\tilde{K}} & = & -\Gamma_{k}diag\left(V^{i}\right)diag\left(V^{i}\right)\tilde{K} \end{array}$$

and the time derivative of the Lyapunov function (A4) along the trajectories of (A7) becomes

(A8) $$\begin{array}{ccc} {\dot{\nu }}_{K} & = & -{\tilde{K}}^{⊤}\Gamma_{k}diag\left(V^{i}\right)diag\left(V^{i}\right)\tilde{K} \end{array}$$

then, from Assumption A1 and selecting $\Gamma_{k}$ as a positive-definite matrix, it follows that the estimation error $\tilde{K}$ converges asymptotically to zero. The last step in this proof is to prove that the interconnection term between the estimator and observer dynamics given by

(A9) $$\begin{array}{ccc} \Psi & = & -\Gamma_{k}diag\left({\tilde{V}}^{i}\right)diag\left({\tilde{V}}^{i}\right)\tilde{K}+2\Gamma_{k}diag\left(V^{i}\right)diag\left({\tilde{V}}^{i}\right)\tilde{K}\\ & & -\Gamma_{k}\left(diag\left(V^{i}\right)-diag\left({\tilde{V}}^{i}\right)\right)diag\left({\tilde{V}}^{i}\right)K\\ & & +\Gamma_{k}diag\left(y_{1}\right)\Gamma_{v}\bar{H}{\tilde{V}}^{i} \end{array}$$

grows lineally with respect to $\tilde{K}$. From, Assumption A2 one has

(A10) $$\begin{array}{ccc} \Psi & \leq & \lambda_{M}\left(\Gamma_{k}\right)\left(∥{\tilde{V}}^{i}{∥}^{2}+2\kappa_{0}∥{\tilde{V}}^{i}∥\right)∥\tilde{K}∥+\lambda_{M}\left(\Gamma_{k}\right)\left(\kappa_{0}+∥{\tilde{V}}^{i}∥)\right)∥{\tilde{V}}^{i}∥∥K∥\\ & & +\lambda_{M}\left(\Gamma_{k}\right)\kappa_{1}\lambda_{M}\left(\Gamma_{v}\right)\lambda_{M}\left(\bar{H}\right)∥{\tilde{V}}^{i}∥ \end{array}$$

The inequality (A10) shows that the interconnection term Ψ grows linearly with respect to the estimation error $\tilde{K}$; thus, Assumption 4.5 of [31] is satisfied. As a result, the cascade system (46) and (24) is asymptotically stable. Hence, the proof is concluded. □
